# Smooth muscle cell sheet transplantation preserve cardiac function and minimize cardiac remodeling in a rat myocardial infarction model

**DOI:** 10.1186/s13019-016-0508-x

**Published:** 2016-08-05

**Authors:** Shingo Harada, Yoshinobu Nakamura, Suguru Shiraya, Yoshikazu Fujiwara, Yuichiro Kishimoto, Takeshi Onohara, Yuki Otsuki, Satoru Kishimoto, Yasutaka Yamamoto, Ichiro Hisatome, Motonobu Nishimura

**Affiliations:** 1 Division of Organ Regeneration Surgery, Department of Surgery, Tottori University Faculty of Medicine, 36-1 Nishi-cho, Yonago, 683-8504 Japan; 2 Division of Regenerative Medicine and Therapeutics, Department of Genetic Medicine and Regenerative Therapeutics, Tottori University Graduate School of Medical Science, Yonago, Japan

**Keywords:** Cell sheet, Smooth muscle cells, Myocardial infarction, Cell survival

## Abstract

**Background:**

We examined whether a vascular smooth muscle cell (SMC) sheet is effective in the treatment of a rat myocardial infarction (MI) model.

**Methods:**

We examined the effect of SMC sheet on the cardiac function and cardiac remodeling in a rat MI model in comparison with their effect of dermal fibroblast (DFB) sheet in vivo. Furthermore, we estimated the apoptosis and secretion of angiogenic factor of SMC under hypoxic condition in comparison with DFB. Seven days after MI, monolayer cell sheets were transplanted on the infarcted area (SMC transplantation group, SMC-Tx; DFB transplantation group, DFB-Tx; no cell sheet transplantation group, Untreated; neither MI nor cell sheet transplantation group, Sham). We evaluated cardiac function by echocardiogram, degree of cardiac remodeling by histological examination, and secretion of angiogenic growth factor by enzyme immunoassay.

**Results:**

Twenty-eight days after transplantation, SMC-Tx showed the following characteristics compared with the other groups: 1) significantly greater fractional area shortening (SMC-Tx, 32.3 ± 2.1 %; DFB-Tx, 23.3 ± 2.1 %; untreated, 25.1 ± 2.6 %), 2) suppressed left ventricular dilation, smaller scar expansion, and preserved wall thickness of the area at risk and the posterior wall, 3) decreased fibrosis, preserved myocardium in the scar area, and greater number of arterioles in border-zone, 4) tight attachment of SMC sheets on the scarred myocardium, and less apoptotic cell death. In in vitro experiments, SMCs secreted higher amounts of basic fibroblast growth factor (SMC, 157.7 ± 6.4 pg/ml; DFB, 3.1 ± 1.0 pg/ml), and showed less apoptotic cell death under hypoxia.

**Conclusions:**

Our results illustrate that transplantation of SMC sheets inhibited the progression of cardiac remodeling and improve cardiac function. These beneficial effects may be due to superior SMC survival.

## Background

Severe cardiac dysfunction after myocardial infarction (MI) impairs the quality of life and leads to poor disease prognosis. Recently, cell transplantation using several cell sources can prevent cardiac dysfunction after MI and dilated cardiomyopathy (DCM) in animal models and in clinical trials: bone marrow mononuclear cells [1], mesenchymal stem cells [2] and skeletal myoblasts [3]. The high attrition rate of transplanted cells [4], due to cell injury through isolation procedure, exposure to ischemic conditions, apoptosis, inflammation, and immunological rejection, is the major limitation of cell transplantation. Increasing the transplanted cell survival rate is paramount to improve the therapeutic potential of this method, since sustained paracrine effects from grafted cells is considered to be a principal mechanism of cell therapy [5].

Cell sheet technology has been developed using temperature-responsive culture dishes. Using this method, cells are harvested from dishes easily by a simple temperature reduction [6]. Recovered cells consist of intact sheets along with the extracellular matrix. Cell sheet methodology improves cell survival compared to cell injection methods [7]. Skeletal myoblast and adipose-tissue derived mesenchymal stem cell sheets attenuate cardiac remodeling in rat MI models [8, 9]. Recently, the autologous skeltal myoblast cell sheets became commercially available for the treatment of ischemic cardiomyopathy in Japan [10]. Ideal characteristics of cells for cell transplantation into post MI tissue are: secretion of angiogenic factors, ease of culture and survival in the host myocardium. Smooth muscle cells (SMCs) secrete several angiogenic factors, such as basic fibroblast growth factor (bFGF) [11], vascular endothelial cell growth factor (VEGF) [12], and hepatocyte growth factor (HGF) [13]; thus a paracrine angiogenic effect is expected. Additionally, it is easy to culture and maintain SMCs compared to other cell types such as endothelial cells or endothelial progenitor cells [14]. SMC transplantation has been conducted in rat MI [4, 15–17] and hind limb ischemia models [13]. However, SMC sheet transplantation for MI has not yet been investigated. In the present study, we examine whether a SMC sheet is effective in the prevention of cardiac dysfunction and remodeling in a rat MI model.

## Methods

We examined the effect of SMC sheet on the cardiac function and cardiac remodeling in a rat MI model in comparison with their effect of DFB sheet in vivo. Furthermore, we estimated the apoptosis and secretion of angiogenic factor of SMC under hypoxic condition in comparison with DFB.

### Experimental animals

Adult male syngenic Lewis rats were obtained from Japan SLC, Inc (Hamamatsu, Japan). Animals weighing 200 to 250 g served as cell donors and recipients. The experimental protocols were approved by the Institutional Animal Care and Use Committee, Faculty of Medicine, Tottori University, and conform to the Guide for the Care and Use of Laboratory Animals published by the US National Institutes of Health (NIH Publication No. 85–23, revised 1996). All the procedures were performed in accordance with the Tottori University animal care guidelines.

### Myocardial infarction

Rats were anesthetized with inhalation of Isoflurane (Abbott Japan, Osaka, Japan), followed by intubation and mechanical ventilation (SN-480-7, Shinano, Tokyo, Japan) at a rate of 60 cycles per minutes with a tidal volume of 2 mL under room air supplemented with oxygen (2 L/min) and 1–2.5 % Isoflurane. The heart was exposed through a 2 cm left thoracotomy, and an anterior myocardial infarction was created by ligation of the proximal coronary artery [left anterior descending artery (LAD)] as previously described [18]. After LAD ligation, the animals recovered from the operation and were given Procaine Penicillin G (Procaine Penicillin G sol, Kawasakimitaka, Kawasaki, Japan) for three days after the operation.

### Preparation of cell sheets

SMCs were isolated from rat aortas. Briefly, descending aorta was isolated, and cut into small pieces (1 × 1 mm) in phosphate buffered saline (PBS). Ten to fifteen pieces of tissue were suspended in Dulbecco’s Modified Eagle’s Medium (DMEM) supplemented with 10 % fetal bovine serum (FBS) and antibiotics. After four to five days, SMCs began to proliferate from the tissue margin. Dermal fibroblasts (DFBs) were isolated from ventral skin explants as described previously [9].

**Fig. 1:**
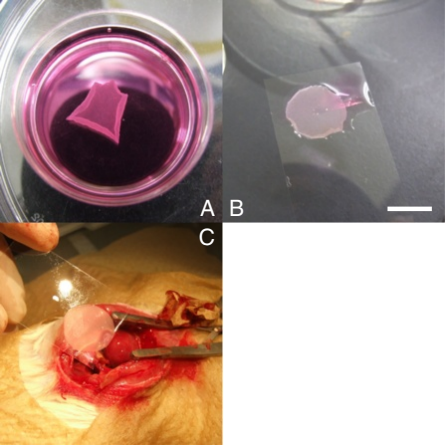
Representative pictures of cell sheet harvesting and transplantation. **a**; Cell sheet which was completely removed from temperature-responsive culture dish (35-mm diameter). Cell sheet shrinks into about 15-mm diameter. Scale bar = 10 mm. **b**; Cell sheet was put on a plastic sheet before transplantation. **c**; The cell sheet was applied face down onto the surface of the anterior scar. The plastic sheet was then carefully removed, leaving the monolayered cell sheet on the surface of the heart

Third passage cells were employed for cell sheet transplantation and in vitro studies. To prepare cell sheets, cells were cultured on 35-mm temperature-responsive culture dishes (UpCell, CellSeed Inc., Tokyo, Japan) at 37 °C in a humidified atmosphere containing 5 % CO_2_. To trace the cells of the transplanted cell sheets, monolayer cell sheets were stained with PKH26 red fluorescent cell linker kit (Sigma-Aldrich Co. St. Louis, MO) for 5 min before cell seeding. After seven days, the cultured cells were transferred to 20 °C for one hour to release the cultured cells as intact sheets (Fig. 1-a). Cell sheet shrinks into about 15-mm diameter (Fig. 1-b, c).

### Transplantation of cell sheets

Surgical mortality of MI operation was 9.1 % (3 of 33 rats), and seven days after coronary ligation, survived 30 rats were randomly divided into 3 treatment groups: (1) SMC sheet transplant (SMC-Tx group, *n* = 10); (2) DFB sheet transplant (DFB-Tx group, *n* = 10); (3) no cell sheet transplantation (Untreated group, *n* = 10). Rats were anesthetized with Isoflurane and the heart was exposed through a median sternotomy. The monolayered cell graft was placed on a plastic sheet and applied face down onto the surface of the anterior scar (Fig. 1-b, c). The plastic sheet was then carefully removed, leaving the monolayered cell graft on the surface of the heart. Ten minutes after transplantation, the chest was closed and the animals were treated in the same manner as described after LAD ligation. The Untreated and Sham (without MI, *n* = 10) groups underwent the same operative procedures without cell grafting. There was no death after cell sheet transplantation in each group.

### Measurements of cardiac function

Cardiac function in the SMC-Tx, DFB-Tx and untreated groups was evaluated with echocardiography six days after LAD ligation prior to sheet transplantation (baseline) and twenty-eight days after cell sheet transplantation. The rats (*n* = 10 rats per group) were sedated with Isoflurane, and left parasternal images were taken in the right lateral decubitus position with a 12-MHz transducer (LOGIQ P5J and 12 L, respectively, GE Healthcare, Fairfield, CT). Short-axis two-dimensional images at the midpapillary level of the left ventricle were stored as digital loops, and the end-systolic (ESA) and end-diastolic (EDA) cavity areas and dimensions were determined by tracing the endocardial borders. The fractional area shortening (FAS) was calculated as (EDA - ESA)/EDA × 100. The ejection fraction (EF) was calculated using Teichholz equation. For each measurement, three consecutive cardiac cycles were traced and averaged by an experienced examiner in a blinded fashion, according to the American Society for Echocardiology Leading Edge Method.

### In vitro measurements of angiogenic factor secretion

In MI, the occlusion of a coronary artery blocks the oxygen and nutrient supply to the myocardium it supplied, and causes severe stress as well as cell death. Transplanted cells face the same severe surroundings. SMCs or DFBs (*n* = 5 per each groups) were seeded on 35 mm dishes and were incubated for four days. Cells were then incubated in normal (37 °C, 5 % CO_2_) or hypoxic conditions for forty-eight hours with 10 % FBS DMEM. Hypoxic conditions (<2 % O_2_) were prepared using a Gaspak system (Becton Dickinson, Bedford, MA). The hypoxic condition was verified by the color of anaerobic indicator which was inside the jar in all experiments. Enzyme-linked immunosorbent assay kits for rat VEGF (Quantikine, R&D, Minneapolis, MN), human bFGF (R&D) and rat HGF (Institute of Immunology, Osaka, Japan) were used according to the manufacturer’s protocols.

### Flowcytometric analysis

Numbers of SMCs isolated from aorta were measured by flowcytometer using anti-α smooth muscle actin antibody (Sigma-Aldrich Co. St. Louis, MO), and PE-conjugated anti-mouse IgG (Becton Dickinson, Bedford, MA). And 98 % of the isolated cell was positive for smooth cell marker (data not shown).

### In vitro apoptosis assay

SMCs or DFBs (*n* = 5 per each groups) were seeded on 35 mm dishes and were incubated for four days. Cell were then incubated in hypoxic conditions using a Gaspak system for forty-eight hours with 10 % FBS DMEM. Cells were then detached with trypsin and apoptotic cell numbers were measured by flow cytometry (ApoAlert Annexin V-FITC Apoptosis Kit, Clontech, Mountain View, CA).

### Planimetric analysis of ventricular morphology

At twenty-eight days after cell sheet transplantation, formalin-fixed hearts (*n* = 5 rats per group) were cut into 2-mm-thick sections, and quantified using digital image analysis (Adobe Photoshop, Adobe Systems Inc., San Jose, CA, and ImageJ, NIH, MD). The left ventricular chamber volume was calculated from planimetric measurements. The thickness of the left ventricular free wall and the scar area were also measured. Briefly, hearts were fixed at a ventricular pressure of 30 mmHg with 10 % phosphate-buffered formalin solution for forty-eight hours. The formalin fixed hearts were cut into 2-mm-thick sections and paraffin embedded. Sections were then stained with Van Gieson staining, and each section were digitally photographed. The area of the left ventricular chamber and length of scar were measured in each section. The ventricular area and scar length was multiplied by the 2 mm thickness of each section, and the sum of all sections was calculated as the left ventricular chamber volume and scar area.

### Histological examination

At twenty-eight days after cell sheet transplantation, the LV myocardium (*n* = 5 from each group) was fixed in 10 % formalin, cut transversely, embedded in OCT compound (Tissue-Tek, Sakura Finetek Japan Co., Ltd, Tokyo, Japan), sliced (10 μm thickness) and stained.

To detect interstitial fibrosis in cardiac muscle, specimens were stained with van Gieson. Transverse sections were obtained from the 3 parts (lateral, posterior, and septum). Three randomly selected fields per section (*n* = 9 per animal) were analyzed at a magnification of 100x (Eclipse-TE200, Nikon, Tokyo, Japan). Each field was scanned and digital images were analyzed (Adobe Photoshop and ImageJ). Collagen composition (red staining) was calculated as the sum of all areas containing connective tissue divided by the total area of the image.

To measure the ratio of preserved myocardium in the risk area, transverse sections were obtained and photographed. The risk area was composed of the elastic fiber depicted by blue signal, scar depicted by red signal and preserved myocardium depicted yellow signal, in Fig. 3-a. We defined the proportion of preserved myocardium in the risk area as the ratio of area depicting yellow signal in the risk area of myocardial infarction. The total area at risk (scar area) and preserved myocardium were scanned and digitally analyzed; the ratio was calculated as (total area of preserved myocardium/total area of risk area) × 100.

To detect microvessels characterized by α-smooth muscle actin (α-SMA) in the border-zone of myocardium and scar, immunohistochemical staining was performed with a monoclonal antibody against α-SMA. Transverse sections were obtained, and 5 of 10 randomly selected fields per animal were analyzed. The number of vessels was counted by light microscopy at a magnification of 200x. The number of arterioles in each field was averaged and expressed as the number of arteriole vessels.

These studies were performed by 2 examiners who were blinded to the sample groups.

### In vivo apoptosis assay

Forty-eight hours after cell sheet transplantation, the number of grafted sheets containing fragmented DNA in the SMC-Tx and DFB-Tx groups (*n* = 5 rats per group) was detected by in situ terminal deoxynucleotidyl transferase-mediated dUTP nick-end labeling (TUNEL) assay using an in situ cell death detection kit according to the manufacturer’s instructions (TACS-XL in situ Apoptosis Detection Kit, TREVIGEN, Gaithersburg, MD). The sample sections were counterstained with Methyl Green. Three microscopic fields (at 200x) of each slide were randomly selected and digitally photographed. To identify the grafted sheets, the sections adjacent to the section used for TUNEL staining were also prestained with the PKH before transplantation. The number of grafted and apoptotic cells were counted separately with digital image analysis (Adobe Photoshop, Adobe Systems Inc., San Jose, CA, and Image J, NIH, MD). Apoptosis was expressed as the number of apoptotic grafted nuclei per field.

### Measurements of cell sheet strength

To measure cell sheet strength, we used a custom-made apparatus. In a water bath perfused with 37 °C physiological saline, SMC and DFB sheets (*n* = 3 per groups) were affixed to a table made from acrylic resin containing a 6 mm hole. Small weights were placed on the cell sheets and digital images were taken. Images were collected at increasing weights (5 – 10 – 20 – 30 – 50 – 100 – 200 – 300 mg), and the maximum tolerance weight was recorded. Additionally, cell sheet displacement was measured (Fig. 6).

### Data analysis

All data are expressed as mean ± SE. One-way analysis of variance (ANOVA) was used for multiple group comparisons. If the F-distribution was significant, a Tukey’s test was used to specify differences between groups. A probability value of < 0.05 was considered significant.

## Results

### Cardiac function: echocardiography

**Fig. 2:**
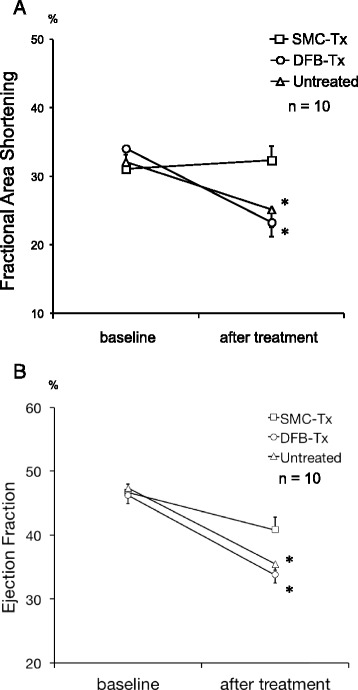
Fractional area shortening (Panel **a**) and ejection fraction (Panel **b**) assessed by echocardiography in experimental groups before cell sheet transplantation (baseline) and twenty-eight days after cell sheet transplantation (after treatment). *p < 0.05 vs. SMC-Tx. SMC-Tx, smooth muscle cell sheet transplantation group; DFB-Tx, dermal fibroblast sheet transplantation group; Untreated, untreated group. Each groups *n* = 10

The final cell counts for monolayered SMCs and DFBs before transplantation were 3.1 ± 0.2 × 10^6^ and 1.5 ± 0.9 × 10^6^ cells, respectively (*n* = 6 each). There was no difference among the three groups in the FAS and EF prior to cell sheet transplantation. Twenty-eight days after cell sheet transplantation, we found a significantly greater FAS in the SMC-Tx group (32.3 ± 2.1 %) compared to the other groups (DFB-Tx, 23.3 ± 2.1 %; untreated, 25.1 ± 2.6 %). Also, we found a significantly greater EF in the SMC-Tx group (40.8 ± 2.0 %) compared to the other groups (DFB-Tx, 33.7 ± 1.2 %; untreated, 35.5 ± 1.0 %). There was no significant difference in the FAS and EF between the DFB-Tx and untreated groups (Fig. 2).

### Ventricular morphology

Morphological assessments of the left ventricle are summarized in Table 1. At twenty-eight days after transplantation, left ventricular volume was significantly smaller (*p* < 0.05) in the SMC-Tx group compared to the DFB-Tx and untreated groups. Similarly, the scar area was significantly (*p* < 0.05) smaller in the SMC-Tx group than in the other groups. Furthermore, wall thickness in the scar area and posterior wall were significantly greater (*p* < 0.05) in the SMC-Tx group, compared to the other groups.

**Table 1:** Ventricular morphology and fibrosis

Group	LV volume (mm^3^)	Scar area (mm^2^)	Scar thickness (mm)	PW thickness (mm)	Fibrosis (%)
Sham	335.1 ± 16.5	–	–	1.87 ± 0.09	1.26 ± 0.16
SMC-Tx	402.9 ± 5.5*	49.4 ± 2.3	1.09 ± 0.05	1.85 ± 0.04	0.87 ± 0.18
DFB-Tx	497.0 ± 24.2*^,^**	83.1 ± 7.4**	0.89 ± 0.05**	1.57 ± 0.06*^,^**	2.33 ± 0.26*^,^**
Untreated	489.9 ± 18.7*^,^**	86.6 ± 11.8**	0.76 ± 0.06**	1.46 ± 0.07*^,^**	2.22 ± 0.35*^,^**

Summary of LV morphological data by planimetry and fibrosis of non-infarcted myocardium collected for each group at twenty-eight days after cell sheet transplantation. *LV* left ventricular, *PW* posterior wall, *SMC-Tx* smooth muscle cell sheet transplantation group, *DFB-Tx* dermal fibroblast sheet transplantation group, *Untreated*, untreated group. Each groups *n* = 5.*p<0.05 vs. Sham, **p<0.05 vs. SMC-Tx.

### Histological findings

**Fig. 3:**
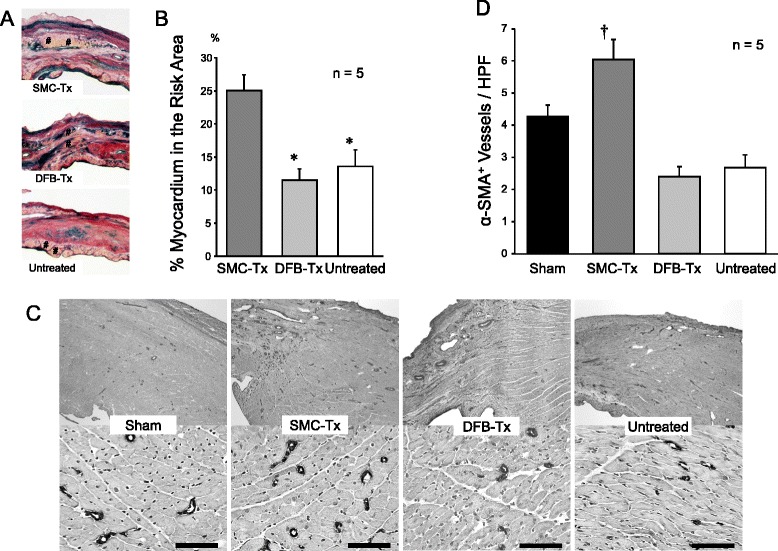
Representative pictures of myocardium (**a**, shown by #) in the scar area of SMC-Tx, DFB-Tx and untreated. **b**; % myocardium in the risk area was significantly higher in SMC-Tx. **c**; Representative pictures of α-SMA^+^ arterioles in border-zone between myocardium and scar area. Upper panels are less magnified pictures of border-zone, and lower panels are higher magnified pictures of border-zone. Scale bar = 100 μm. D; α-SMA^+^ arterioles in border-zone were significantly increased in SMC-Tx. *p < 0.05 vs. SMC-Tx; †*p* < 0.05 vs. other groups. SMC-Tx, smooth muscle cell sheet transplantation group; DFB-Tx, dermal fibroblast sheet transplantation group; Untreated, untreated group. Each groups *n* = 5

Twenty-eight days after treatment, Van Gieson staining revealed significantly less fibrosis in the SMC-Tx group than in the other groups (Table 1). Additionally, the SMC-Tx group showed more preserved myocardium in the risk area compared to the other groups (Fig. 3-a, b). In the SMC-Tx group, there were a greater number of mature vessels in the border-zone compared to the DFB-Tx and untreated groups (Fig. 3-c, d).

**Fig. 4:**
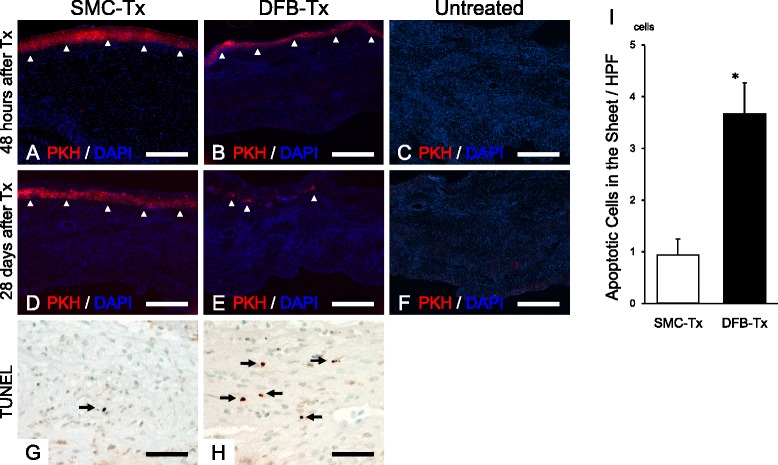
Confirmation of the attachment of transplanted cell sheet at forty-eight hours (**a**–**c**) and twenty-eight days (**d**–**f**) after transplantation. Left panels are SMC-Tx (**a**, **d**), middle panels are DFB-Tx (**b**, **e**), and right panels are Untreated (**c**, **f**). Red signal, PKH stained cell sheet; blue signal, DAPI for nuclear staining. Microphotographs of transplanted cell sheet forty-eight hours after transplantation that has been stained with TUNEL assay in SMC-Tx (**g**) and DFB-Tx (**h**) groups. Numbers of TUNEL-positive grafted cell sheets (**i**). *p < 0.01 vs. SMC-Tx. Each groups *n* = 5. Scale bar = 500 μm (**a**–**f**), 50 μm (**g**, **h**). SMC-Tx, smooth muscle cell sheet transplantation group; DFB-Tx, dermal fibroblast sheet transplantation group; Untreated, untreated group

Forty-eight hours after cell sheet transplantation, SMC and DFB sheets were confirmed to exist on the infarcted areas as layers (Fig. 4-a, b). Twenty-eight days after transplantation, SMC sheets existed on the scar as a layer (Fig. 4-d). In contrast, DFB sheets were scattered (Fig. 4-e). There was no red fluorescence in the untreated group at both forty-eight hours and twenty-eight days after transplantation (Fig. 4-c, f).

We observed whether PKH26-fluorescent marker positive cells migrate into the ischemic zone in all the samples. However, no migrated cell was detected in our observation.

### Cell survival in vitro and in vivo

**Fig. 5:**
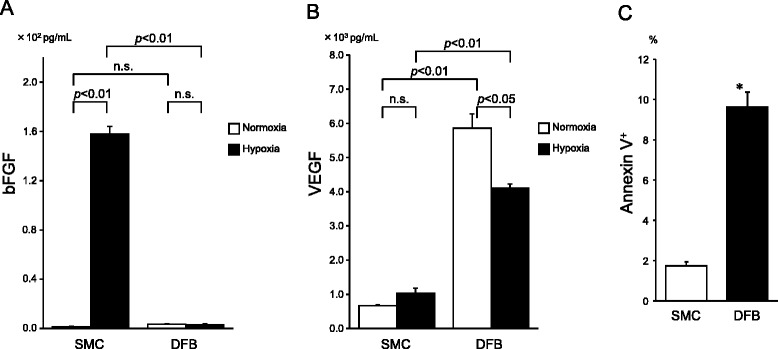
Angiogenic factor secretion under normal and hypoxic condition in vitro. **a**; bFGF. **b**; VEGF. **c**; Apoptosis assay of cells cultured in vitro in hypoxic conditions. *p < 0.01 vs. SMC. Each groups *n* = 5

The number of apoptotic cells was significantly lower in hypoxic SMCs (1.7 ± 0.2 %) compared to DFBs (9.6 ± 0.8 %) in vitro (Fig. 5-c). Additionally, TUNEL-positive SMCs were rarely detected (0.9 ± 0.3cells/HPF) in the sheets 48 h after transplantation. In contrast, TUNEL-positive DFBs were frequently detected (3.7 ± 0.6cells/HPF) in the DFB sheets (Fig. 4-g, h, i).

### Cell sheet strength

**Fig. 6:**
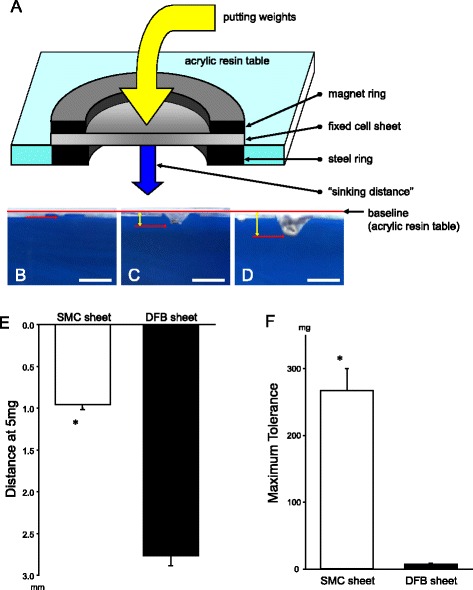
Representative schema of custom-made apparatus to measure cell sheet strength (**a**). Cell sheets were stretched by weights and photographed. “Sinking Distance” was then measured (*blue arrow*). Representative pictures of stretched cell sheet by weight loading to measure cell sheet strength (**b**–**d**). SMC sheet at 5 mg (**b**), DFB sheet at 5 mg weight (**c**) and SMC sheet at 200 mg (**d**). “Sinking Distance” was measured as a distance within *red bars* and *red arrows* (*yellow bicephalic arrows*). Scale bar (*white bar*) = 5 mm. Sinking distance at 5 mg (**e**) and maximum weight tolerance (**f**) was measured. *p < 0.05 vs. DFB sheet. SMC, smooth muscle cell; DFB, dermal fibroblast. Each groups *n* = 3

SMC sheets showed significantly less sinking distance with the 5 mg weight than DFB sheets (SMC sheet, 0.96 ± 0.06 mm; DFB sheet, 2.77 ± 0.12 mm, *p* < 0.05) (Fig. 6-b, c, e). Similarly, SMC sheets showed significantly higher maximum weight tolerance (266.7 ± 33.3 mg) compared to DFB sheets (6.7 ± 1.7 mg, *p* < 0.05) (Fig. 6-d, f).

### Secretion of angiogenic factors

bFGF production in SMCs and DFBs cultured in normoxic conditions were very low and comparable; however, bFGF significantly increased in SMC (normoxia, 1.3 ± 0.5 pg/ml; hypoxia, 157.7 ± 6.4 pg/ml) but not DFB (normoxia, 3.6 ± 0.5 pg/ml; hypoxia, 3.1 ± 1.0 pg/ml) cultured in hypoxia (Fig. 5-a). In contrast, DFBs secreted about four-fold higher VEGF in hypoxic conditions (4112 ± 114 pg/ml), and about nine-fold higher VEGF in normoxic conditions (5857 ± 418 pg/ml) than SMCs (normoxia, 665 ± 33 pg/ml; hypoxia, 1029 ± 148 pg/ml) (Fig. 5-b). HGF were not detected in normoxic and hypoxic conditions in both SMC and DFB culture medium.

## Discussion

Our results indicate that SMC sheet transplantation preserved cardiac function and prevented cardiac remodeling compared to DFB sheet transplantation. These effects correlated with the prevention of hypoxia-induced apoptosis, production of bFGF, and increased mechanical support of the infarcted myocardium in a rat MI model. To the best of our knowledge, this is the first report that investigated the effect of SMC sheet transplantation in a rat MI model.

To escape from cell death and dysfunction in ischemic condition, blood perfusion recovery and obtain the tolerance against ischemia may deal effectively. In hypoxic conditions, SMCs showed a significantly lower number of apoptotic cells than DFBs in vitro (Fig. 5-c). Additionally, forty-eight hours after transplantation post MI we observed less apoptotic cells in the SMC-Tx group compared to the DFB-Tx group (Fig. 4-g, h, i) in vivo. Because SMCs have a higher tolerance for ischemia and hypoxia, SMC sheets may be able to survive on the scar as an attached layer (Fig. 4-d) at twenty-eight days after transplantation. In contrast, DFB sheets could not survive in vivo. It has been reported that preventing apoptosis of transplanted cells can increase cell survival and ventricular function [17], and loss of transplanted cell leads to deterioration of cardiac function in a mouse model [5]. These concepts suggest that the superior survival rate of transplanted cells is essential for cell therapy.

It is known that bFGF stimulates the maturation of vasculature, which are lined with vascular smooth muscle cells. These vessels could provide long-term stable perfusion [19]. SMCs secreted significantly higher amounts of bFGF in hypoxic conditions compared to SMCs in normoxia and hypoxic DFBs (Fig. 5-a). Our data suggest that bFGF secreted by SMC sheets increased arteriole number in the border-zone between healthy myocardium and the scar areas (Fig. 3-c, d), which was consistent with previous report [20, 21] and maintained viable myocardium in the scar area (Fig. 3-a, b). In contrast, DFBs secreted a higher amount of VEGF than SMCs in hypoxic and normoxic conditions (Fig. 5-b). VEGF is widely known as one of the critical factors in promoting angiogenesis [20]; however, DFB sheets did not improve cardiac function [9]. Our data suggest that DFB sheets could not survive hypoxia and thus could not produce sufficient amounts and duration of VEGF to rescue cardiomyocytes although VEGF could be a more potent angiogenic factor than bFGF. Thus, we considered that the higher tolerance of SMC sheets to hypoxia enables them to survive longer after transplantation. Furthermore, the long-term secretion of bFGF by transplanted SMC sheets might contribute to maintain arterioles and may support the survival of the SMC sheet cells and the myocardium in the late phases. One possible explanation is that SMCs are secreting more potent angiogenic factor than VEGF, which we did not evaluate. The other possibility is that the paracrine effect of SMCs last longer although the angiogenic effect itself is inferior.

SMC sheet transplantation preserved scar thickness in vivo (Table 1). Preserving scar thickness is very important to reduce wall stress [9, 22] and prevent ventricular dilatation. Additionally, cardiac support devices, which are designed as mesh-like implantable devices that are surgically positioned around the heart and adjusted to provide circumferential diastolic support and prevent ventricular dilatation, have been used [23, 24]. Because SMC sheets showed significantly higher weight tolerance than DFB sheets (Fig. 6), we considered that SMC sheets contributed mechanical support to prevent scar thinning and expansion in the weakened myocardium. On the other hand, it is expected that using artificial materials as a patch may contribute to reinforcement; however, it is difficult to uniformly attach the artificial materials onto the scar. In contrast, attachment of biological tissue onto the scar is feasible, and the secretion of angiogenic factors has been reported [8, 9, 25]. For these reasons we considered that transplantation of the SMC sheet could be a promising candidate for future clinical applications.

Cell sheet transplantation of skeletal myoblasts was reported to have advantages over cell injection methods in MI and DCM models [8, 26, 27]. Cell sheet technology enables the transplantation of cells with more physiological conditions, and the cell loss compared with direct cell injection is lower. Similar properties could be expected with SMC sheets [13]. Cell sheet technology has been investigated not only in heart failure, but also in the treatment of other organ dysfunctions, such as cornea [28], periodontal ligament [29], oral mucosa [30] and urothelium [31]; therefore future development is expected.

We investigated only up to thirty-five days after MI; however, investigation for much longer periods will be required before the clinical application of SMC sheets can be conducted. The feasibility of SMC sheets may be controversial; however, our data support beneficial effects. Further technical advances are expected using embryonic stem cells and induced pluripotent stem cells in the near future; therefore these technologies might help investigators to supply many different cell sources.

## Conclusions

SMC sheets contribute to cardiac function and attenuate cardiac remodeling with sufficient effects of angiogenic factors which are brought through superior cell survival. Additionally, SMC sheets seem to contribute to cardiac function by serving as mechanical support for the infarcted myocardium. These results indicate that this method may be a new strategy for ischemic cardiomyopathy therapy.

## References

[1] Nishida M, Li TS, Hirata K, Yano M, Matsuzaki M, Hamano K (2003). Improvement of cardiac function by bone marrow cell implantation in a rat hypoperfusion heart model. Ann Thorac Surg.

[2] Nagaya N, Kangawa K, Itoh T (2005). Transplantation of mesenchymal stem cells improves cardiac function in a rat model of dilated cardiomyopathy. Circulation.

[3] Jain M, DerSimonian H, Brenner DA (2001). Cell therapy attenuates deleterious ventricular remodeling and improves cardiac performance after myocardial infarction. Circulation.

[4] Yasuda T, Weisel RD, Kiani C, Mickle DA, Maganti M, Li RK (2005). Quantitative analysis of survival of transplanted smooth muscle cells with real-time polymerase chain reaction. J Thorac Cardiovasc Surg.

[5] Ziebart T, Yoon CH, Trepels T (2008). Sustained persistence of transplanted proangiogenic cells contributes to neovascularization and cardiac function after ischemia. Circ Res.

[6] Yang J, Yamato M, Kohno C (2005). Cell sheet engineering: recreating tissues without biodegradable scaffolds. Biomaterials.

[7] Shimizu T, Yamato M, Kikuchi A, Okano T (2003). Cell sheet engineering for myocardial tissue reconstruction. Biomaterials.

[8] Memon IA, Sawa Y, Fukushima N (2005). Repair of impaired myocardium by means of implantation of engineered autologous myoblast sheets. J Thorac Cardiovasc Surg.

[9] Miyahara Y, Nagaya N, Kataoka M (2006). Monolayered mesenchymal stem cells repair scarred myocardium after myocardial infarction. Nat Med.

[10] Sawa Y, Yoshikawa Y, Toda K (2015). Safety and efficacy of autologous skeletal myoblast sheets (TCD-51073) for the treatment of severe chronic heart failure due to ischemic heart disease. Circ J.

[11] Ali S, Becker MW, Davis MG, Dorn GW (1994). Dissociation of vasoconstrictor-stimulated basic fibroblast growth factor expression from hypertrophic growth in cultured vascular smooth muscle cells. Relevant roles of protein kinase C. Circ Res.

[12] Stavri GT, Zachary IC, Baskerville PA, Martin JF, Erusalimsky JD (1995). Basic fibroblast growth factor upregulates the expression of vascular endothelial growth factor in vascular smooth muscle cells. Synergistic interaction with hypoxia. Circulation.

[13] Hobo K, Shimizu T, Sekine H, Shin’oka T, Okano T, Kurosawa H (2008). Therapeutic angiogenesis using tissue engineered human smooth muscle cell sheets. Arterioscler Thromb Vasc Biol.

[14] Asahara T, Murohara T, Sullivan A (1997). Isolation of putative progenitor endothelial cells for angiogenesis. Science.

[15] Li RK, Jia ZQ, Weisel RD, Merante F, Mickle DA (1999). Smooth muscle cell transplantation into myocardial scar tissue improves heart function. J Mol Cell Cardiol.

[16] Liu TB, Fedak PW, Weisel RD (2004). Enhanced IGF-1 expression improves smooth muscle cell engraftment after cell transplantation. Am J Physiol Heart Circ Physiol.

[17] Nakamura Y, Yasuda T, Weisel RD, Li RK (2006). Enhanced cell transplantation: preventing apoptosis increases cell survival and ventricular function. Am J Physiol Heart Circ Physiol.

[18] Matsubayashi K, Fedak PW, Mickle DA, Weisel RD, Ozawa T, Li RK (2003). Improved left ventricular aneurysm repair with bioengineered vascular smooth muscle grafts. Circulation.

[19] Atluri P, Liao GP, Panlilio CM (2006). Neovasculogenic therapy to augment perfusion and preserve viability in ischemic cardiomyopathy. Ann Thorac Surg.

[20] Taguchi A, Soma T, Tanaka H (2004). Administration of CD34+ cells after stroke enhances neurogenesis via angiogenesis in a mouse model. J Clin Invest.

[21] Isner JM, Asahara T (1999). Angiogenesis and vasculogenesis as therapeutic strategies for postnatal neovascularization. J Clin Invest.

[22] Wang T, Wu DQ, Jiang XJ (2009). Novel thermosensitive hydrogel injection inhibits post-infarct ventricle remodelling. Eur J Heart Fail.

[23] Oz MC, Konertz WF, Kleber FX (2003). Global surgical experience with the Acorn cardiac support device. J Thorac Cardiovasc Surg.

[24] Blom AS, Mukherjee R, Pilla JJ (2005). Cardiac support device modifies left ventricular geometry and myocardial structure after myocardial infarction. Circulation.

[25] Sekine H, Shimizu T, Hobo K (2008). Endothelial cell coculture within tissue-engineered cardiomyocyte sheets enhances neovascularization and improves cardiac function of ischemic hearts. Circulation.

[26] Hamdi H, Furuta A, Bellamy V (2009). Cell delivery: intramyocardial injections or epicardial deposition? A head-to-head comparison. Ann Thorac Surg.

[27] Kondoh H, Sawa Y, Miyagawa S (2006). Longer preservation of cardiac performance by sheet-shaped myoblast implantation in dilated cardiomyopathic hamsters. Cardiovasc Res.

[28] Nishida K, Yamato M, Hayashida Y (2004). Functional bioengineered corneal epithelial sheet grafts from corneal stem cells expanded ex vivo on a temperature-responsive cell culture surface. Transplantation.

[29] Akizuki T, Oda S, Komaki M (2005). Application of periodontal ligament cell sheet for periodontal regeneration: a pilot study in beagle dogs. J Periodontal Res.

[30] Hayashida Y, Nishida K, Yamato M (2005). Ocular surface reconstruction using autologous rabbit oral mucosal epithelial sheets fabricated ex vivo on a temperature-responsive culture surface. Invest Ophthalmol Vis Sci.

[31] Shiroyanagi Y, Yamato M, Yamazaki Y, Toma H, Okano T (2004). Urothelium regeneration using viable cultured urothelial cell sheets grafted on demucosalized gastric flaps. BJU Int.

